# Efficient monocular 3D lane detection via Mamba-enhanced CM-3DLane framework

**DOI:** 10.1038/s41598-026-44870-1

**Published:** 2026-03-26

**Authors:** Yilin Yang, XinChen Zhang, Ying Liu

**Affiliations:** 1 College of Physical and Technology, Central China Normal University, Luoyu Road, Wuhan, 430070 Hubei China; 2 Wollongong joint Institute, Central China Normal University, XiongChu Road, Wuhan, 430070 Hubei China

**Keywords:** 3D lane detection, Visual state space model, Feature fusion, Autonomous driving, Engineering, Mathematics and computing

## Abstract

Monocular 3D lane detection provides richer spatial information than 2D lane detection planar positioning results. It is crucial for enhancing vehicle perception in complex intelligent driving scenarios. Recent advances primarily model lanes in 3D space via anchor lines, project them onto front-viewed (FV) features for sampling, and directly regress 3D coordinates from 2D image features. However, the slender structural attributes of lanes pose significant challenges for accurate localization within 3D space. Existing frameworks struggle with effectively integrating multi-level features to capture global spatial structural relationships essential for detection accuracy and face difficulties in balancing detection performance with computational efficiency. To alleviate these problems, we present a novel CM-3DLane framework, an efficient 3D lane detector. Instead of directly superimposing deeper and lower-level features, we propose a strategy for multi-scale information integration that exploits a convolutional neural network (CNN) backbone for extracting local image features. We propose the Lane-Aware Mamba (LAMamba) block, which employs a tailored 2D selective scan (SS2D) strategy. This enables linear-complexity modeling of long-range spatial dependencies and global lane context, significantly enhancing feature extraction. This is complemented by a Cross-Scale Attention Fusion (CSAF) module that leverages channel and spatial attention mechanisms to effectively fuse multi-scale features. In addition, we design a Refined Anchor Dynamic Ranking (RADR) module to preserve the most representative and informative 3D anchors. CM-3DLane scores 58.3 F1 on OpenLane and 96.5 F1 on ApolloSim, leading all prior methods while maintaining high efficiency suitable for real-time deployment.

## Introduction

3D lane detection is pivotal for autonomous-driving functionalities such as trajectory planning ^1^ and lane keeping. Although LiDAR-based approaches have achieved notable success in other 3D perception tasks ^2^, recent 3D lane-detection research ^3^ increasingly favors monocular cameras because of their inherent advantages over LiDAR sensors. In addition to lower deployment costs, cameras provide an extended perception range and deliver high-resolution images rich in texture–attributes that are essential for accurately detecting slim, long-range lanes.

The absence of explicit depth cues renders 3D lane recovery from monocular imagery inherently arduous. A prevalent remedy is to cast the problem into the bird-eye-viewed (BEV). Standard BEV-based pipelines ^4^ first employ inverse perspective mapping (IPM) to transform either raw images or extracted features from the FV to BEV, effectively reducing the 3D detection objective to a 2D lane localization task in the BEV plane. Subsequently, the 2D BEV lane points are lifted back to 3D space by augmenting their planar coordinates with per-point elevation estimates produced by a dedicated height-prediction module. While this strategy has demonstrated empirical efficacy, several notable shortcomings persist. First, IPM depends heavily on the flat ground assumption, which becomes invalid for uphill or downhill scenarios, resulting in misaligned 3D coordinate estimations of lane lines under such road conditions. Second, IPM performs the perspective transformation based on the ground plane, which inevitably loses valuable clues like height and context information above the road’s surface. In addition, objects on the road such as vehicles may undergo severe distortion after IPM, causing the lane markings to be occluded.

To avoid the above limitations that hinder the accuracy of 3D information restoration from BEV representations. Recent advances such as Anchor3DLane ^5^ abandons the BEV paradigm. It structurally models lanes in 3D space via anchor lines, projects them onto FV features for sampling, and directly regresses 3D coordinates from 2D image features. However, compared to BEV space, a significant gap persists between FV space and the 3D representation of lane markings. The slender structural characteristics of lanes further complicate precise 3D localization. Consequently, fusing multi-scale features from FV imagery to capture global spatial relationships is critical for enhancing lane detection accuracy.

Effectively dealing with the scale variations of lanes across different road sections, the lane detection model focuses on both the detailed parts (e.g., endpoints, bifurcations of lanes) and the global aspects (e.g., the overall shape of the lanes, curvature variations, etc.). Smaller scale lanes require fine-grained feature extraction, while larger scale lanes require the network to be able to capture the macroscopic road structure. Recently, CNNs such as ResNet ^6^ and EfficientNet ^7^, limited receptive field constrains their ability to model broad-scope irregular dependencies across the entire image, resulting in discontinuous lanes and weak background noise suppression. Although dilated convolutions ^8^ expand the receptive field, their inherent inductive bias still prevents them from fully addressing this issue ^9^, especially in complex road curvatures and geometries with heavy background interference. The success of Vision Transformer (ViT) ^10^ has demonstrated Transformer’s effectiveness in capturing irregular pixel dependencies, which is crucial for recognizing complex lanes textures. However, the quadratic complexity of attention calculations with sequence length leads to high memory use and training challenges for high-resolution images, limiting deployment on resource-constrained edge devices and practical applications. In conclusion, in the practical application of autonomous driving, the algorithms must not only ensure accuracy but also meet lightweight requirements. Especially at high speeds or in complex road environments, the system must be able to quickly sense and make decisions about lane information to ensure safety ^11^.

**Fig. 1:**
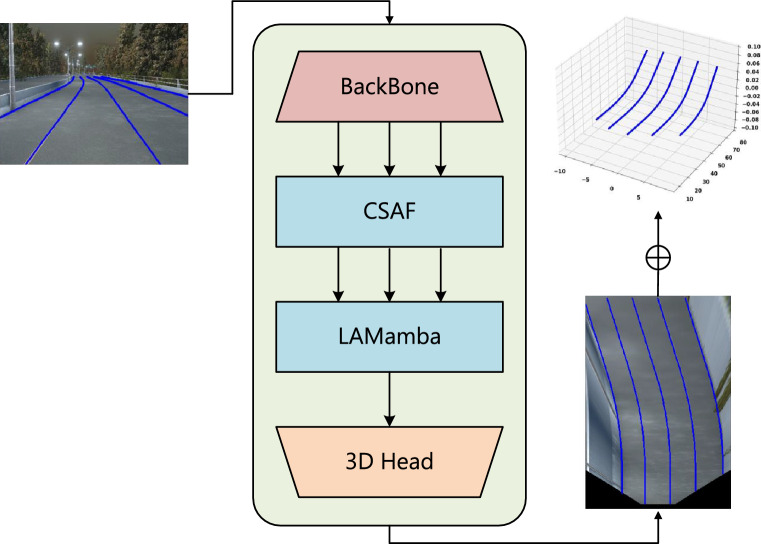
First, the input images are fed into the backbone network to extract multi-scale features. Next, the Cross-Scale Attention Fusion module and Lane-Aware Mamba module perform deep processing of these features to generate FV features. Finally, the FV features are passed to the 3D Head for prediction, and the results are further processed to obtain the 3D lane detection output.

To tackle the challenge of balancing high 3D lane detection quality with low computational demands, we design CM-3DLane, an efficient and concise 3D lane detection method built upon Anchor3DLane ^5^. It defines lane anchors as rays in the 3D space with given yaws and pitches. As shown in Fig. 1, CNN Backbone first performs multi-level feature extraction to fully explore the rich information contained in different network depths and feature sizes. Then inspired by Vision Mamba (ViM) ^12^ and VMamba ^13^ have extended Mamba to the visual domain, which attracts considerable interest due to Mamba’s showing strong performance in sequence modeling while maintaining low computational demands ^14^. We design a Lane-Aware Mamba block(LAMamba) for effectively integrating information across various scales extracted by the backbone network. Specifically, the LAMamba block utilizes the Mamba block for capturing long-range dependencies with linear time complexity and domain features enhance spatial consistency and improves detail processing in lane detection. Moreover, a Cross-Scale Attention Fusion (CSAF) module is introduced to enable CNN backbone multi-scale features to move beyond purely parallel processing and dynamically adapt across different scales, aiding the LAMamba block through skip connections. By combining with the attention module, more robust and high-level features can be extracted, further enhancing the overall performance of the network. Furthermore, we also design a Refined Anchor Dynamic Ranking (RADR) module to preserve the most representative and informative 3D anchors. Finally, a multi-output supervision strategy is applied across different decoder stages in CM-3DLane, guiding the network to progressively refine its predictions for high-accuracy 3D lane detection.

While recent anchor-based methods like Anchor3DLane offer a promising direction by directly modeling 3D anchors, they primarily rely on CNN backbones for feature extraction. This reliance limits their ability to effectively model the long-range spatial dependencies and global context that are crucial for accurate 3D localization of slender, often discontinuous lane structures, especially under complex road geometries. Conversely, Vision Transformers (ViTs), though adept at capturing global context, suffer from quadratic computational complexity, making them less suitable for real-time applications on resource-constrained platforms.

To address these specific gaps, we present CM-3DLane, a novel framework designed not merely as an integration of existing modules, but as a domain-specific architectural co-design tailored to the unique topological properties of lane markings. The core theoretical contribution of our work lies in rethinking how State Space Models (SSMs) interact with the geometric continuity of lanes. Unlike generic Vision Mamba implementations that use standard scanning paths, we propose a novel “Diagonal Snake Scan” strategy within our Lane-Aware Mamba (LAMamba) block. We theoretically demonstrate that standard parallel or simple snake scans disrupt the visual continuity of curved lanes, whereas our diagonal serpentine path aligns with the natural evolution of lane trajectories, enabling linear-complexity modeling of global topology without breaking structural coherence.

Furthermore, our novelty extends to a tripartite synergy: (1) The CSAF module is explicitly designed to preserve fine-grained texture cues often lost in generic multi-scale fusion, which is critical for distinguishing faint lanes from complex road surfaces; (2) The RADR module introduces a dynamic sparsification mechanism driven by semantic priors and geometric constraints, fundamentally reducing representation ambiguity rather than simply selecting anchors. Comprehensive evaluations confirm that this specialized design achieves a new state-of-the-art balance, delivering superior accuracy (58.3% F1 on OpenLane) while maintaining real-time efficiency (78 FPS), thus validating the effectiveness of our domain-adapted Mamba architecture over generic alternatives.

## Related work

### 2D lane detection

2D lane detection ^15–19^ seeks to accurately delineate the geometric form and spatial position of lanes within an image. Earlier works ^20–24^ predominantly relied on extracting low-level handcrafted features. Such strategies, however, necessitate intricate extraction pipelines and delicate post-processing, and their robustness deteriorates when confronted with variable environments. The advent of deep learning has motivated recent studies to adopt CNNs, yielding substantial performance gains.

Segmentation-based frameworks ^25–27^ formulate 2D lane detection as a semantic/instance segmentation task and the key to these methods is the development of more efficient and semantically rich feature extraction techniques. To achieve sparser and more flexible predictions, keypoint-based methods ^28–31^ represent lane markings as ordered sets of salient points and then aggregate keypoints belonging to the same lane through subsequent grouping procedures. Different from these bottom-up methods above, anchor-based methods ^32,33^ use predefined anchors to model lanes and subsequently regress the offsets between the sampled points and the anchors, enabling 2D lane prediction. Additionally, 2D lanes are modeled by introducing row anchors in the row direction and grid cells in the column direction, which significantly enhances the accuracy and efficiency of lane detection. Despite a certain degree of progress in 2D lane detection techniques, there is still a non-negligible gap between the 2D results and the requirements of real-world applications. Especially in complex road environments, 2D detection results provide insufficient spatial information. To bridge this gap, the 3D lane detection technology approach has received attention. It can better understand and model road geometry in 3D space, thus providing more accurate 3D lane location.

### 3D lane detection

Since projecting 2D lanes into 3D space introduces substantial inaccuracies and reduced robustness, the community has shifted toward 3D lane detection. Early endeavors leverage multi-modal sensing, including stereo rigs ^34^ and LiDAR–camera setups ^35^, to recover depth. Nevertheless, the high expenses associated with multi-sensor data acquisition and annotation limit their real-world deployment. Consequently, methods that infer 3D lanes solely from monocular imagery ^36–39^ have garnered increasing interest.

Among these methods, transforming Front-View (FV) representations to Bird’s-Eye-View (BEV) has been a prevalent solution due to the superior geometric properties of lanes in BEV space. Early works like 3DLaneNet ^4^ and Gen-LaneNet ^37^ utilized Inverse Perspective Mapping (IPM) for this transformation. However, IPM’s reliance on the flat-ground assumption leads to significant misalignments on uneven terrain. Recent advances have sought to mitigate this: CLGo ^38^ incorporates camera pose estimation to refine BEV transformation, while SALAD ^39^ decomposes the task into 2D segmentation and depth estimation to bypass direct IPM limitations. More recently, Transformer-based approaches such as CurveFormer ^40^ and LATR ^41^ have adopted DETR-like architectures to implicitly model 3D lanes via query embeddings, achieving robust performance but at the cost of high computational overhead.

To further enhance feature representation, dual-view and prior-guided methods have emerged. DV-3DLane ^42^ proposes a bidirectional fusion strategy between PV and BEV spaces, utilizing a unified query generator to aggregate dual-view features. Similarly, PVALane ^43^ introduces prior-guided view-agnostic feature alignment to fuse geometric and semantic information. However, a critical limitation persists across these BEV and Transformer-based SOTA methods: their computational complexity scales quadratically with sequence length or requires heavy multi-view synchronization, hindering deployment on edge devices. While efficient architectures like MobileViT ^44^ and EfficientViT^45^ have shown promise in general vision tasks, their application to 3D lane detection remains underexplored, particularly in balancing the need for long-range dependency modeling with strict latency constraints.

In contrast to these approaches, our method unlocks the potential of Mamba-based State Space Models specifically for 3D lane detection. Unlike generic efficient backbones, our CM-3DLane framework performs feature perception and fusion at multiple scales with linear complexity, explicitly addressing the trade-off between global semantic understanding and local texture preservation. By integrating a tailored SS2D mechanism, we achieve superior spatial coherence and detailing capabilities, offering a distinct advantage over both heavy Transformer models and limited-receptive-field CNNs in complex, large-scale driving scenes.

### State space models

Linear State-Space Models (SSMs) ^46,47^ have emerged as a computationally efficient alternative to CNNs and Transformers for modeling long sequences. The Structured State Space Sequence Model (S4)  ^14^first demonstrated the efficacy of linear state-space contextualization, achieving competitive results across diverse benchmarks. Subsequent diagonal approximations–HTTYH ^48^, DSS ^49^, and S4D ^50^–reduced computational complexity while preserving accuracy. The S5 ^51^ further advanced practical applicability by introducing a parallel scan operation and a Multiple-Input–Multiple-Output (MIMO) SSM formulation. More recently, Mamba ^52^ proposed a selective SSM coupled with a hardware-aware training algorithm, enabling linear-time inference and scalable training. In the vision domain, Vision Mamba ^12^ leverages bidirectional state-space representations to compress visual features, whereas VMamba ^13^ generalizes 1-D selective scanning to 2-D images, endowing the model with global receptive fields. Owing to its versatility, Mamba has subsequently been adopted in a broad range of applications, including graph learning ^53^, medical image segmentation ^54^, video understanding ^55^, and generative modeling ^56^.Furthermore, Mamba’s selective state space model has also been shown to effectively improve performance in image classification tasks ^57^.

Currently, no high-performing Mamba-based model exists for lane detection. Thus, designing an optimized VSS structure specifically for lane detection is essential to improve performance and efficiency. Given the intricate details and irregular textures of lanes, the VSS block requires strong shape extraction and directional awareness to effectively capture lane texture cues. Additionally, it should facilitate efficient capture of detailed lane features while minimizing computational resource requirements.

### SS2D

SS2D is recently advanced by Liu et al. ^13^, which augments Mamba to handle two-dimensional visual inputs instead of one-dimensional textual sequences. Its core mechanism, termed Cross-Scan, employs four-directional rasterization to construct the state-space model. Specifically, the input image is decomposed into patches and traversed along four distinct paths, producing four independent sequences that are subsequently processed by the S6 (also known as Mamba) block. As a backbone, SS2D attains global receptive fields while maintaining linear computational complexity.

## Proposed method

**Fig. 2:**
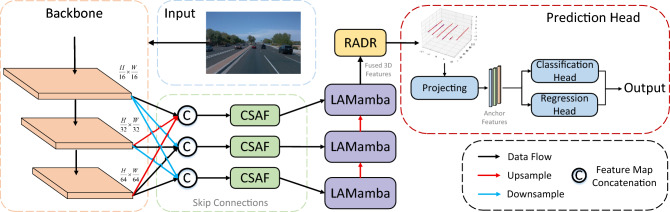
The overall architecture of CM-3DLane. The framework follows a clear modular design: (1) The CSAF module is responsible for fusing multi-scale features from the backbone through dedicated channel and spatial attention mechanisms, enhancing the representation of lanes at different scales. (2) The LAMamba neck then processes these refined features. Its core component, the SS2D, efficiently models global contextual and structural relationships across the entire scene, which is essential for coherent 3D lane detection.

The overall architecture of our CM-3DLane is illustrated in Fig. 2. Firstly, we use a CNN backbone to extract the multi-scale feature mapping $X \in \mathbb {R}^{C \times H \times W}$ from the input FV image, where H and W denote the height and width of the image respectively. We then process the multi-scale features using the CSAF Module. It fuses low-level and high-level feature information to enhance the perception of lanes at different scales. In the LAMamba neck, the sequence is processed through the LAMamba block, embedding key lanes pixel cues into multiscale feature maps $\{F^1, F^2, F^3\}$ with a comprehensive semantic understanding. $F^3$ is first fed into the RADR module to obtain refined 3D anchors $A_j$, which offer essential positional priors contextualized by the input image. Finally, we apply the prediction header to the enhanced FV features to obtain the final lane prediction.

### Backbone

We adopt ResNet ^6^ as the backbone network for the CM-3DLane feature extraction process. ResNet is a landmark model in the history of deep learning. It solves model degradation problems in deep networks by using shortcut connections. Using ResNet, we can extract multi-scale features and generate feature maps with $\frac{1}{16}, \frac{1}{32} \text { and } \frac{1}{64}$ resolution, thus capturing rich spatial information at different scales and enhancing the perceptual capability of the model.

### Cross-scale attention fusion module

The performance of CNN-based detectors is often constrained by a fundamental architectural trait: scale sensitivity. This limitation arises from the fixed receptive field of convolutional kernels, which hinders their adaptability to objects of vastly different sizes. While this affects general object detection, it poses a unique challenge in lane detection. Here, the visual scale of a lane is not intrinsic but is dramatically influenced by its distance from the camera and the road’s geometry, causing extreme scale variation within a single image.

**Fig. 3:**
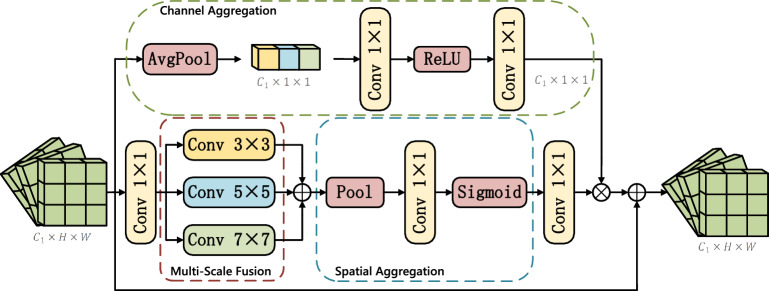
The structure of Cross-scale Attention Fusion module.

Existing cross-scale fusion modules, designed for broader object categories, typically prioritize large-scale contextual integration. However, they often overlook the fine-grained textual and structural details that are paramount for distinguishing lanes from the road surface and avoiding false positives. In complex driving scenarios, where lighting, occlusion, and road conditions vary, these limitations directly contribute to increased omission and commission errors, highlighting the need for a more specialized approach. To address these challenges, we propose a novel Cross-Scale Attention Fusion Module. Following the established methodology in attention mechanisms ^58,59^, the CSAF adopts a two-branch structure, the upper branch focuses on multi-scale feature extraction and aggregation in the spatial domain, while the lower branch handles feature extraction and aggregation in the channel domain. Finally, the outputs of these two branches are fused through element-wise multiplication.

Fig. 3 depicts the CSAF module for refining feature representations in FV recognition, highlighting both spatial and channel perception pathways. Formally, outputs from ResNet backbone stages, $C_1 \in \mathbb {R}^{C \times H \times W}, C_2 \in \mathbb {R}^{C \times H \times W}, \text {and } C_3 \in \mathbb {R}^{C \times H \times W}$, are concatenated along the channel dimension. Then the combined features $\hat{X}$ are subsequently processed by the CSAF for refinement. This module employs a dual-path architecture dedicated to spatial and channel-wise feature aggregation. The spatial refinement path first utilizes a 1$\times$1 convolution for channel projection to reduce dimensionality. It then performs multi-scale feature fusion by summing the outputs of convolutional layers with kernel sizes of 3$\times$3, 5$\times$5, and 7$\times$7. Subsequently, mean and max pooling operations are applied to aggregate spatial features. Finally, a 7$\times$7 convolution is executed, and its output undergoes element-wise multiplication with the sigmoid-activated feature map. The detailed operations in spatial paths can be formulated as follows:

1 $$\begin{aligned} \hat{X} \in \mathbb {R}^{C_x \times H \times W}= & \text {Concat}(C_1, C_2, C_3) \end{aligned}$$

2 $$\begin{aligned} X'_s=  \text {ReLU}(W_s^1 * \hat{X}) \end{aligned}$$

3 $$\begin{aligned} X_s''=  \sum _{k \in \{3, 5, 7\}} \text {Conv}_{k \times k}(X_s') \end{aligned}$$

4 $$\begin{aligned} A_s=  \text {Sigmoid}(\text {Conv}_{7 \times 7}(\text {AvgPool}(X_s'') + \text {MaxPool}(X_s''))) \end{aligned}$$

5 $$\begin{aligned} \hat{X}_s=  A_s \odot X_s'' \end{aligned}$$

Where $W_s^1$ denotes convolution kernel parameters, *r* is a compression ratio and $\odot$ denotes Hadamard product.

In parallel, the channel aggregation path processes the features through global average pooling, reducing the dimensions to $C_1$ $\times$ 1 $\times$ 1. This is subsequently fed through a series of 1$\times$1 convolutions and a ReLU activation function to produce a channel attention map. Ultimately, this map is spatially expanded to the original feature dimensions and fused with the output from the spatial refinement path. The detailed operations in channel paths can be formulated as follows:

6 $$\begin{aligned} z_c= & GA(\hat{X}) \end{aligned}$$

7 $$\begin{aligned} A_c= & ReLU(W_c^2 * \delta (W_c^1 * z_c)) \end{aligned}$$

8 $$\begin{aligned} \tilde{X}_s= & A_c \otimes \hat{X} \end{aligned}$$

9 $$\begin{aligned} X_{output}= & \tilde{X}_s + \tilde{X}_c \end{aligned}$$

Where GA denotes global average pooling and $\otimes$ denotes matrix multiplication.

The CSAF enriches spatial details and channel-wise dependencies for downstream tasks. The incorporation of the CSAF module serves to significantly enrich the final feature maps with comprehensively refined spatial and channel-based characteristics.

### Lane-aware mamba block

Unlike natural language or general images, lanes can be geometrically viewed as slender, continuous, and topologically structured sequences evolving in space. Accurate detection relies not only on local texture but also on modeling long-range spatial dependencies. The performance of lane detection systems is significantly enhanced by models capable of effectively integrating both global contextual understanding and local detailed processing. In recent years, transformer-based architectures, which leverage self-attention mechanisms, have shown considerable success across a range of computer vision applications. Despite their effectiveness, these models are often constrained by quadratic computational complexity with respect to sequence length, leading to scalability and efficiency issues when handling detailed lane features. This limitation highlights the necessity for novel solutions that preserve the representational power of transformers while alleviating their computational burden.

**Fig. 4:**
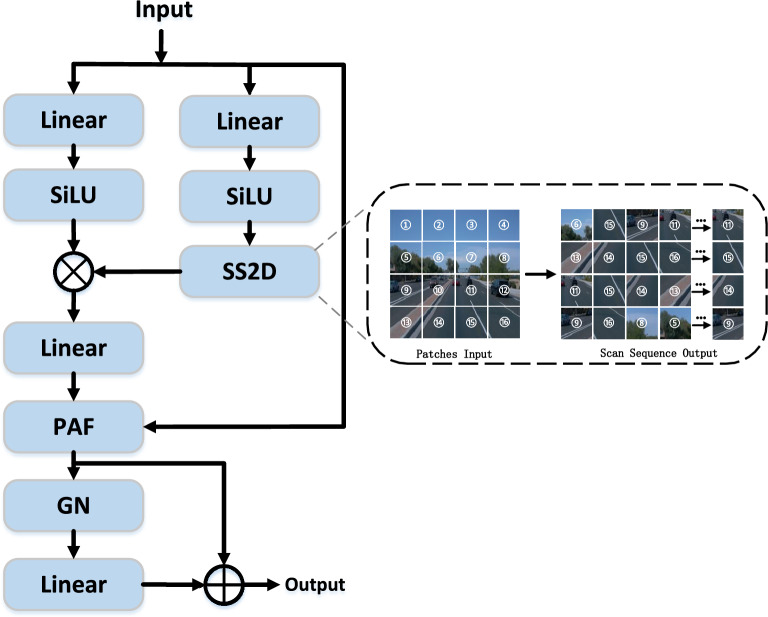
The internal architecture of LAMamba. The SS2D module serves as the core, performing state scans in four directions (horizontal, vertical, and diagonal). The attention mechanisms are integrated as post-processing enhancement layers following SS2D: the output features from SS2D undergo channel-wise reweighting to highlight key feature channels, followed by spatial masking to amplify responses in lane-specific regions.

The ability of State Space Models (SSMs) to model sequence evolution with linear complexity makes them an ideal choice for capturing such global lane geometry.Inspired by the efficiency of Mamba in long-range sequence modeling with linear complexity, we propose the LAMamba module, which reformulates 3D lane detection as a structured state-space modeling problem. The core objective is to leverage the inherent sequential modeling capability of SSM to capture the global topological evolution of lane markings.

As illustrated in Fig. 4, the LAMamba block treats the feature map as a collection of spatial states. To overcome the causality constraint of 1D SSM, we employ the SS2D mechanism. This process maps the input feature *X* into four distinct scanning paths (horizontal, vertical, and their reversals), ensuring that each spatial position can perceive structured information from the entire global context. From a structural perspective, this serves as a global prior that maintains the connectivity of slender lane markings across long distances.

**Fig. 5:**
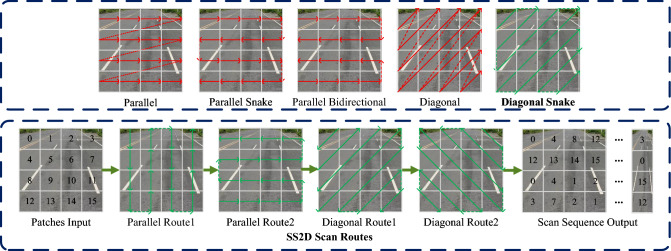
Illustration of our proposed SS2D and other scanning strategies. The first row presents four commonly used single scanning paths, along with our proposed diagonal snake path. The second row illustrates the execution flow of our proposed SS2D scanning strategy.

Different scanning strategies impact the model’s ability to capture continuous lane textures. As shown in Fig. 5, current vision Mamba networks use various scanning directions. Standard SS2D scanning strategies (e.g., parallel, snake, diagonal scans ^12,13^) have scan paths with limited directional continuity, which may break the visual continuity of lane lines that exhibit curved or diagonal extensions during the scanning process. Our proposed Diagonal Snake Scan strategy, by combining diagonal movements with serpentine folding-back, creates a more continuous scan path on the image plane that better conforms to the direction of lane curves, thereby more effectively integrating long-range contextual information of lane pixels and improving modeling of complex lane geometry.

After the FV image undergoes ResNet backbone and CSAF, it is input as a sequence into the LAMamba block. To maintain a lightweight network, we use 3 layers of LAMamba blocks. The processing equations are as follows:

10 $$\begin{aligned} \bar{P}= & \exp (\Delta _t P) \end{aligned}$$

11 $$\begin{aligned} \bar{Q}= & (\Delta _t P)^{-1} (\exp (\Delta _t P) - I) \Delta _t Q \end{aligned}$$

12 $$\begin{aligned} z_k= & \bar{P} \cdot z_{k-1} + \bar{Q} w_k \end{aligned}$$

13 $$\begin{aligned} u_k = R z_k + S w_k \end{aligned}$$

In these equations, the input $w \in \mathbb {R}^{t \times D}$ , $P \in \mathbb {R}^{G \times D}$ controls the hidden spatial state, $S \in \mathbb {R}^{D \times D}$ is used to initialize the skip connection for input, $z_k$ represents the specific hidden state at time step k , and $P \in \mathbb {R}^{G \times D}$ and $R \in \mathbb {R}^{G \times D}$ are matrices with hidden spatial dimensions G and temporal dimensions D , respectively, obtained through selective scanning SS2D. These are trainable parameters that are updated accordingly. $u_k$ represents the output at time step k. SASS establishes multi-directional adjacency relationships, allowing the hidden state $z_k$ to capture more intricate topological and textural details, while enabling the output $u_k$ to more effectively integrate multi-directional features.

To effectively combine the initial sequence *x* with the sequence processed through SS2D, we incorporate Pixel Attention-oriented Fusion (PAF) ^60^, enhancing LAMamba’s ability to capture lane shape and texture details. Following selective scanning, a residual connection is applied to the fused information to preserve detail and facilitate feature flow.

### Representation of 3D lane anchors

**Fig. 6:**
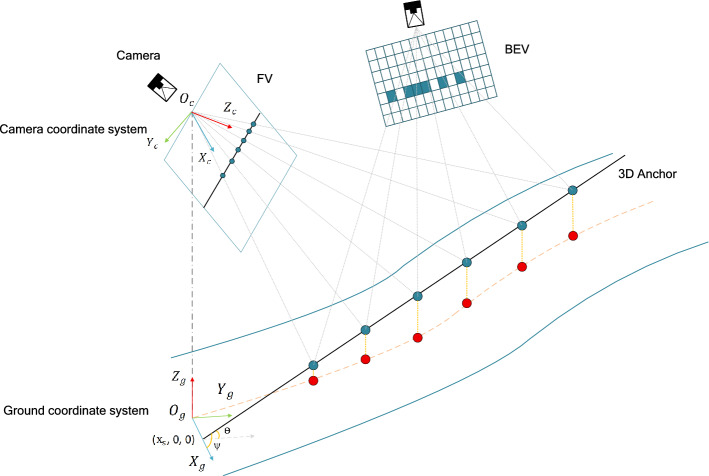
Illustration of 3D anchor and 3D lane in the ground coordinate system.

We utilize the anchor method introduced by AnchorLane for lane representation due to its simplicity and robustness. Our 3D lane anchors are defined within the same ground coordinate system as the 3D lanes themselves, facilitating position regression. As depicted in Fig. 6, a 3D anchor originates from ($x_s$, 0, 0) with pitch $\theta$ and yaw $\varphi$. Consistent with 3D lanes, we sample N points for each anchor at the same y-coordinates. The j-th 3D anchor is represented as $A_j$=$\{p_j^k\}_{k=1}^N$ , with its k-th point denoted by $p_j$= $\{x_j^k, y_j^k, z_j^k\}$. The key advantage of our anchor representation lies in its ability to directly model the 3D geometry of lanes without relying on approximations or transformations that could introduce errors. By aligning the anchors with the ground coordinate system, we ensure that the model can effectively learn the spatial relationships between lanes and their surroundings, leading to more precise detection results. This method provides a solid foundation for the subsequent stages of lane detection, enabling the model to handle the complexities of real-world driving scenarios with improved accuracy and reliability.

### Refined anchor dynamic ranking module

Building on the Anchor3DLane framework, CM-3DLane defines anchor lines within a 3D metric space to facilitate monocular 3D lane detection in the front view. While this approach is conceptually straightforward, it places significant computational demands on the model due to its reliance on dense 3D anchor lines. These demands manifest not only in increased computational cost and extended running time but also in an elevated risk of overfitting. To mitigate these challenges, this study introduces the RADR module. This innovative component employs attention mechanisms and geometric priors to enable intelligent selection of anchor lines.

**Fig. 7:**
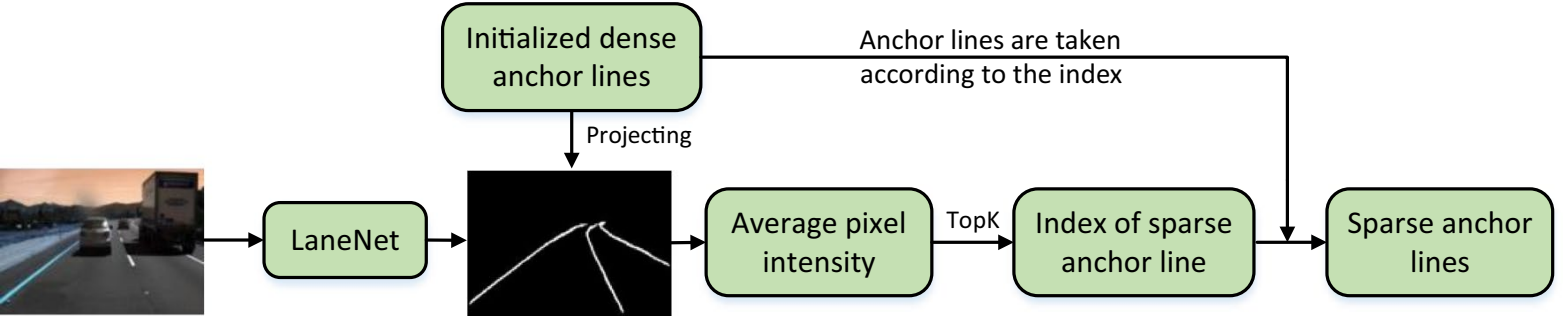
The structure of Refined Anchor Dynamic Ranking Module.

As shown in Fig. 7, The original 2D image is processed using the semantic segmentation network of the 2D lane line detection model LaneNet ^61^ to produce a binary segmentation map M, which serves as a pseudo-label for distinguishing between foreground and background regions of lane lines. The dense 3D anchors $A_j$ are projected onto the segmentation map M via camera intrinsic and extrinsic parameters to obtain the pixel values of all points on the anchor lines. If a point’s projection falls outside the image boundaries, its pixel value is set to zero. For each point $p_i$ , multi-scale features are extracted as follows:

14 $$\begin{aligned} F_i = \begin{bmatrix} M(u_i, v_i) \\ \nabla M(u_i, v_i) \\ C(u_i, v_i) \end{bmatrix} \end{aligned}$$

where $\nabla M$ represents gradient magnitude and *C* represents local contrast. A lightweight MLP then computes spatial-channel attention weights:

15 $$\begin{aligned} W_i = MLP(F_i) \end{aligned}$$

Subsequently, the anchor score $s_k$ is calculated as:

16 $$\begin{aligned} S_k = \frac{1}{N_k} \sum _{i=1}^{N_k} W_i M(u_i, v_i) \end{aligned}$$

This anchor score reflects the intensity of the anchor line on the binary segmentation map and serves as a critical metric for evaluating the anchor line. Anchors are ranked according to $s_k$, with the top-K candidates $\{A_k\}_{k=1}^K$ being selected. To enforce geometric consistency, a regularization term is added to the training loss:

17 $$\begin{aligned} L_s = \lambda _1 \sum _{k=1}^{K-1} ||w_k - w_{k+1}||_2 + \lambda _1 \sum _{k=1}^{K} \left| \left| \frac{\partial ^2 A_k}{\partial s^2}\right| \right| _2 \end{aligned}$$

In this equation, $w_k$ represents the lane width of anchor k, and $\frac{\partial ^2 A_k}{\partial s^2}$ signifies curvature discontinuity. This module offers multiple benefits. It reduces unnecessary computation, enhances the clarity of lane line representation, and improves the model’s prediction accuracy. By retaining the most representative and informative anchor lines, the RADR module ensures efficient and precise lane detection.

### Overall loss function

The total loss function of our CM-3DLane is defined as:

18 $$\begin{aligned} L_{total} = \lambda _{cls} L_{cls} + \lambda _{reg} L_{reg} + \lambda _s L_s \end{aligned}$$

where $\lambda _{cls}$, $\lambda _{reg}$ and $\lambda _{s}$ denote the loss coefficients. The classification loss $\lambda _{cls}$ is calculated using the binary cross-entropy loss. Given the predicted probability $c_j$ for each anchor $A_j$ and its corresponding label $s_j$, the loss is formulated as:

19 $$\begin{aligned} L_{cls} = -\sum _{j=1}^{M_a} \left[ s_j \log (c_j) + (1 - s_j) \log (1 - c_j) \right] \end{aligned}$$

20 $$\begin{aligned} L_{reg} =&\sum _{i=1}^{M_p} \left\| \hat{v}_{is_i} \cdot (x_{\sigma (i)} + \Delta x_{\sigma (i)} - \hat{x}_i) \right\| _1 \nonumber \\&+ \sum _{i=1}^{M_p} \left\| \hat{v}_{is_i} \cdot (z_{\sigma (i)} + \Delta z_{\sigma (i)} - \hat{z}_i) \right\| _1 \nonumber \\&+ \sum _{i=1}^{M_p} \left\| \hat{v}_{is_{\sigma (i)}} - \hat{v}_{is_i} \right\| _1 \end{aligned}$$

where $M_p$ represents the number of positive proposals, and $\sigma (i)$ indicates the index of the proposal assigned to the i-th ground-truth lane.

## Experiments

### Dataset

We conduct experiments on two popular 3D lane detection benchmarks, including ApolloSim ^37^ and OpenLane ^62^.

**ApolloSim** is a synthetic dataset created using the Unity 3D engine with a variety of 10.5K virtual scene images, including highways, cities, homes, and city centers. In addition, the picture data is diverse in terms of daylight, weather conditions, traffic obstacles, and road quality. It is divided into three subsets: 1) Balanced scenes, 2) Rarely observed scenes, and 3) Scenes with visual variations.

**OpenLane** is a comprehensive, large-scale benchmark for 3D lane detection, built on the Waymo dataset ^63^. This dataset consists of 1000 segments, including 200K frames captured under various weather, terrain, and brightness conditions at a resolution of 1280$\times$1920. OpenLane contains 880K lane annotations, which are spread across a total of 14 categories, providing a realistic and diverse set of challenges for 3D lane detection algorithms.

### Evaluation metrics

During the evaluation process, a minimum-cost flow algorithm is adopted to match lane predictions and ground truth lanes and the matching cost is calculated as the square root of the sum of the pointwise Euclidean distance between the sampled points. If more than 75% of the points’ distances between a prediction and a ground-truth lane are below 1.5m, this prediction will be considered as true positive. Utilizing this definition, we evaluate performance using Average Precision (AP) and the maximum F1-score, along with lateral (x) and longitudinal (z) errors at near (0–40 m) and far (40–100 m) ranges. On the ApolloSim dataset, we report the F1 score, AP, and positional errors x/z-errors. For the OpenLane dataset, in addition to the F1 score and positional errors, we further introduce category accuracy, defined as the ratio of correctly classified predictions to all true positive detections.

### Implementation details

All experimental evaluations were performed using an NVIDIA GeForce RTX 2080 Ti graphics processor, with the MMDetection3D framework serving as our primary development platform. We use input size 360×480 and adopt ResNet-18 ^64^ as our backbone to extract feature maps. To preserve feature resolution, we reduce the downsampling stride of the last two stages to 1 and replace standard 3$\times$3 convolutions with dilated convolutions. The starting positions $x_s$ of 3D anchors are initialized along the x-axis at uniform intervals of 1.3 m. For each $x_s$, we define multiple orientations by varying yaw angles $\phi \in \{0^\circ , \pm 1^\circ , \pm 3^\circ , \pm 5^\circ , \pm 7^\circ , \pm 10^\circ , \pm 15^\circ , \pm 20^\circ , \pm 30^\circ \}$ and pitches $\theta \in \{0^\circ , \pm 1^\circ , \pm 2^\circ , \pm 5^\circ \}$ are set respectively. The total number of sparse anchors $N_a$ is set to 30. To balance computational efficiency and detection accuracy, we sample N=20 anchor points for the ApolloSim dataset and N=10 for the OpenLane dataset, determined based on their respective y-coordinate ranges.During training, our implementation utilizes the AdamW optimizer with L2 regularization strength set to $\lambda$=0.01 for parameter optimization. The initial learning rate was set to $2 \times 10^{-4}$ and the cosine annealing scheduler was used. The training protocol comprised 30 epochs on the OpenLane dataset and 80 epochs on ApolloSim, with a consistent batch size of 16. Model selection was performed based on optimal validation metrics, with the top-performing checkpoint subsequently evaluated on test data.

### Comparison studies

#### Results on ApolloSim

**Fig. 8:**
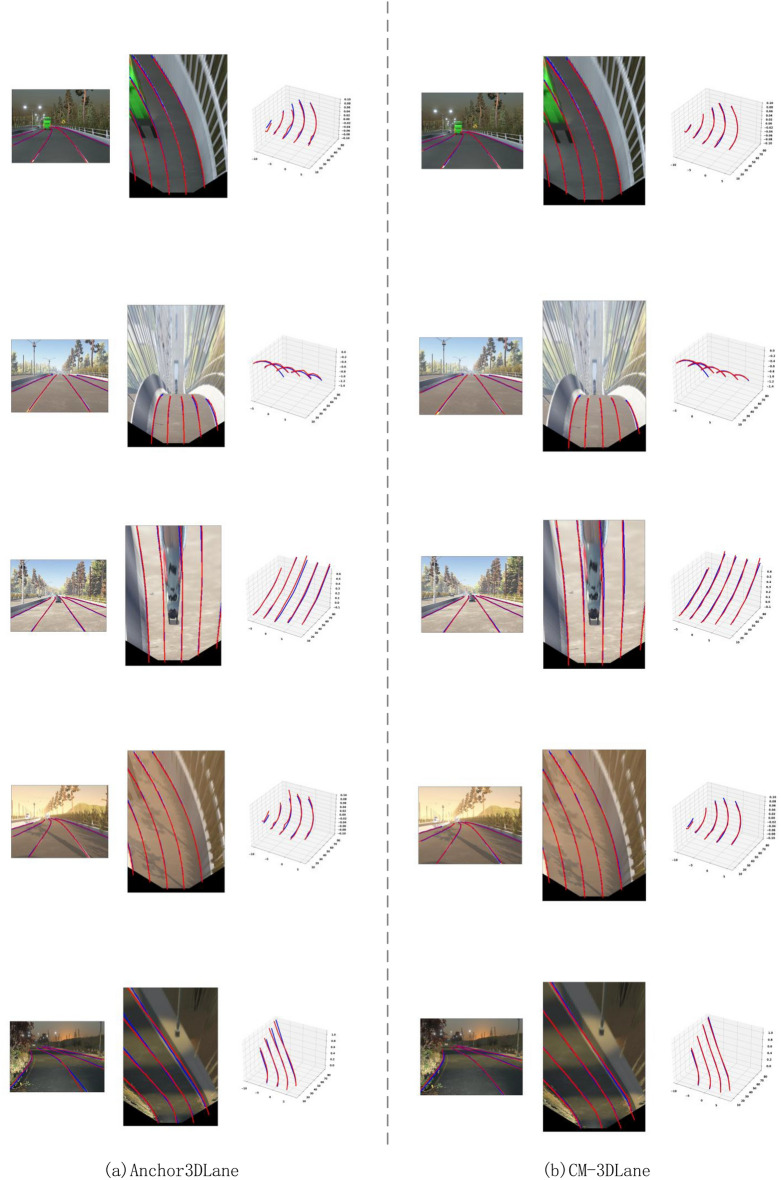
Qualitative comparison on ApolloSim. We choose AnchorLane as our comparison method. We visualize the results in different scenarios. Each visualization includes the FV image (left), the BEV output (middle), and the 3D lanes (right). The blue lanes are the ground truth, the red lanes are the predicted results.

**Table 1:** Result of different models on ApolloSim.

Scene	Method	AP (%)	F1 (%)	x-Err/N (m)	x-Err/F (m)	z-Err/N (m)	z-Err/F (m)
Balanced Scene	3DLaneNet ^4^	89.3	86.4	0.068	0.477	0.015	0.202
	Gen-LaneNet ^37^	90.1	88.1	0.061	0.496	0.012	0.214
	CLGo ^38^	94.2	91.9	0.061	0.361	0.029	0.250
	PersFormer ^65^	–	92.9	0.054	0.356	0.010	0.234
	GP ^37^	93.8	91.9	0.049	0.387	0.008	0.213
	CurveFormer ^40^	97.3	97.3	0.079	0.326	0.018	0.219
	Anchor3DLane ^5^	95.6	95.4	0.052	0.306	0.016	0.223
	Anchor3DLane++ ^66^	97.4	96.0	0.029	0.272	0.015	0.217
	Ours	97.7	96.5	0.031	0.200	0.016	0.208
Rare Subset	3DLaneNet ^4^	74.6	72.0	0.166	0.855	0.039	0.521
	Gen-LaneNet ^37^	79.0	78.0	0.139	0.903	0.030	0.539
	CLGo	88.3	86.1	0.147	0.735	0.071	0.609
	PersFormer ^65^	–	87.5	0.107	0.782	0.024	0.602
	GP ^37^	85.2	83.7	0.126	0.903	0.023	0.625
	CurveFormer ^40^	97.1	95.6	0.182	0.737	0.039	0.561
	Anchor3DLane ^5^	95.9	94.4	0.094	0.695	0.030	0.579
	Anchor3DLane++	96.5	95.3	0.052	0.624	0.027	0.560
	Ours	96.8	96.1	0.042	0.594	0.027	0.544
Visual Variations	3DLaneNet ^4^	74.9	72.5	0.115	0.601	0.032	0.230
	Gen-LaneNet ^37^	87.2	85.3	0.074	0.538	0.015	0.232
	CLGo ^38^	89.2	87.3	0.084	0.464	0.045	0.312
	PersFormer ^65^	–	89.6	0.074	0.430	0.015	0.266
	GP ^37^	92.1	89.9	0.060	0.446	0.011	0.235
	CurveFormer ^40^	93.0	90.8	0.125	0.410	0.028	0.254
	Anchor3DLane ^5^	92.5	91.8	0.047	0.327	0.019	0.219
	Anchor3DLane++ ^66^	95.1	92.4	0.045	0.385	0.021	0.261
	Ours	95.9	93.8	0.036	0.350	0.019	0.229

As presented in Table 1, our model demonstrates strong overall accuracy with an AP of 97.7% in the balanced scene, slightly outperforming leading methods such as CurveFormer ^40^ and baseline Anchor3DLane ^5^. The F1 score of 96.5% further illustrates the precision and recall stability achieved by CM-3DLane. Importantly, our method achieves the lowest z-axis localization error in the far range (z-Err/F) of 0.208m, a notable improvement compared to other methods, emphasizing its effectiveness in distant lane detection. In the rare subset, our method displays its robustness in complex and infrequent lane configurations. Our method achieves an AP of 96.8% and an F1 score of 96.1%. This robustness is further confirmed by our model’s minimal x-axis error in the far range (x-Err/F) of 0.594m and a reduced z-axis error (z-Err/F) of 0.544m, both of which are the lowest among all evaluated methods. These results highlight the resilience of CM-3DLane in scenarios that challenge depth and spatial perception, a key aspect of 3D lane detection. In scenarios with visual variations, which introduce additional challenges due to lighting and environmental changes, our method maintains its advantage with an AP of 95.9% and an F1 score of 93.8%. Our method also minimizes x-axis errors far from the camera (x-Err/F), achieving 0.350m, the lowest across all methods tested. This precision, particularly under visually challenging conditions, suggests that our method effectively retains contextual information and spatial accuracy, even with significant visual disturbances. In addition, we provide qualitative comparison results, as shown in Fig. 8.

#### Results on OpenLane

**Fig. 9:**
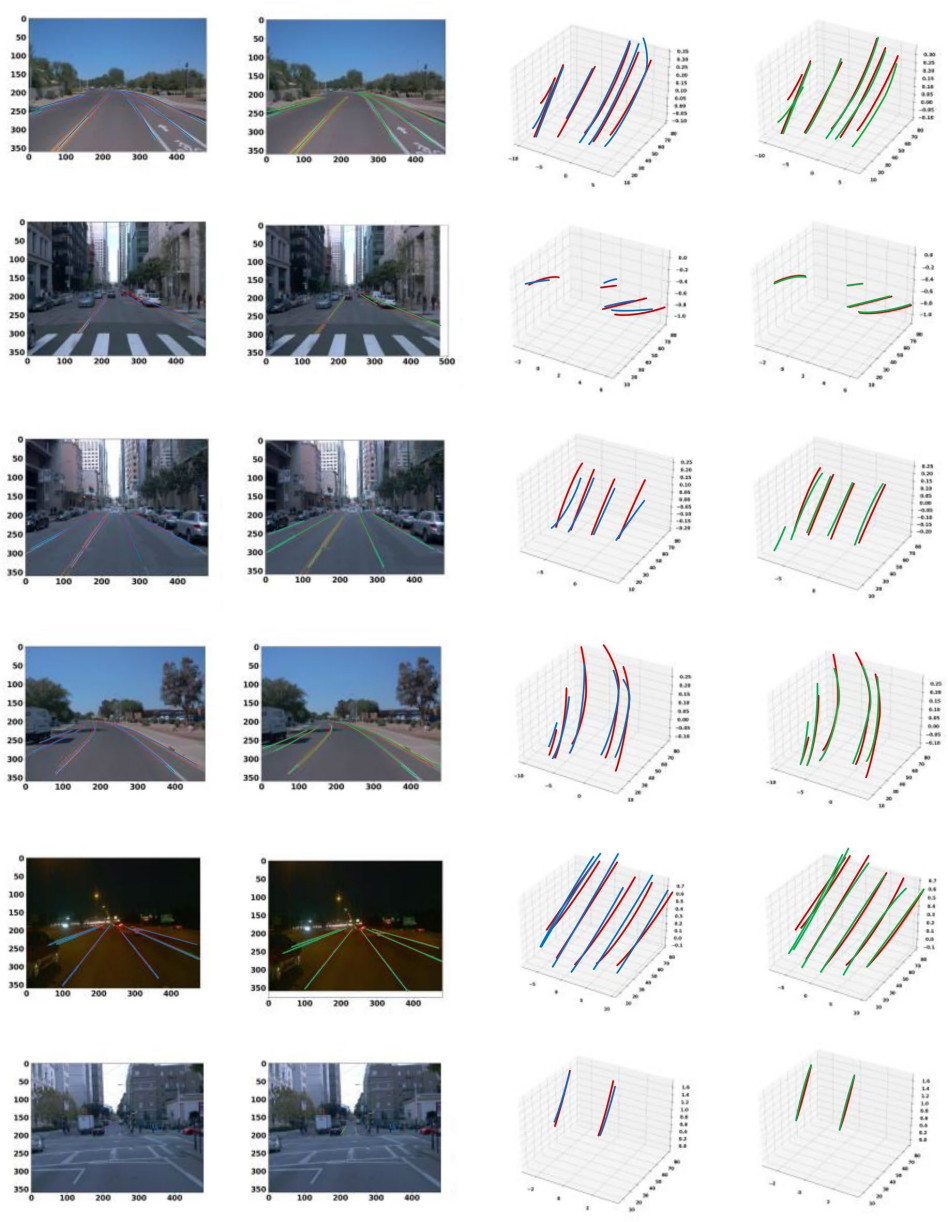
Qualitative comparison results on Openlane dataset. Each result includes the FV image (left) and the 3D lanes (right). The red lanes are the ground truth, the green lanes are the predicted results of our method, and the blue lanes represent the Anchor3DLane output.

Table 2 presents a quantitative comparison of our method against other models across multiple metrics. Notably, our method achieved the highest AP and F1 scores, as well as the lowest x-axis and z-axis errors in both the close and far ranges. Specifically, our model reached an F1 score of 58.3%, surpassing the baseline model, Anchor3DLane. Furthermore, our model demonstrated superior accuracy in spatial localization, as indicated by the minimal x-errors and z-errors at far distances from the camera, reflecting improved robustness in depth and lateral position estimation.In addition to the quantitative results, we show the qualitative visualization results in Fig. 9.

**Table 2:** Comparison with various methods on OpenLane validation set.

Method	F1(%)	Cate Acc(%)	x-Err/N(m)	x-Err/F(m)	z-Err/N(m)	z-Err/F(m)
3DLaneNet ^4^	44.1	–	0.479	0.572	0.367	0.443
Gen-LaneNet ^37^	32.3	–	0.591	0.684	0.411	0.521
PersFormer ^65^	50.5	92.3	0.485	0.553	0.364	0.431
CurveFormer ^40^	50.5	–	0.340	0.772	0.207	0.651
Anchor3DLane ^5^	53.1	90.7	0.300	0.311	0.107	0.345
Ours	58.3	91.8	0.240	0.249	0.196	0.268

Notably, our CM-3DLane method maintains competitive inference speeds while achieving state-of-the-art F1 scores. This balance makes CM-3DLane particularly well-suited for real-time scenarios. The method thus offers a strong balance of performance and efficiency, demonstrating its potential for deployment in demanding real-time applications.

**Fig. 10:**
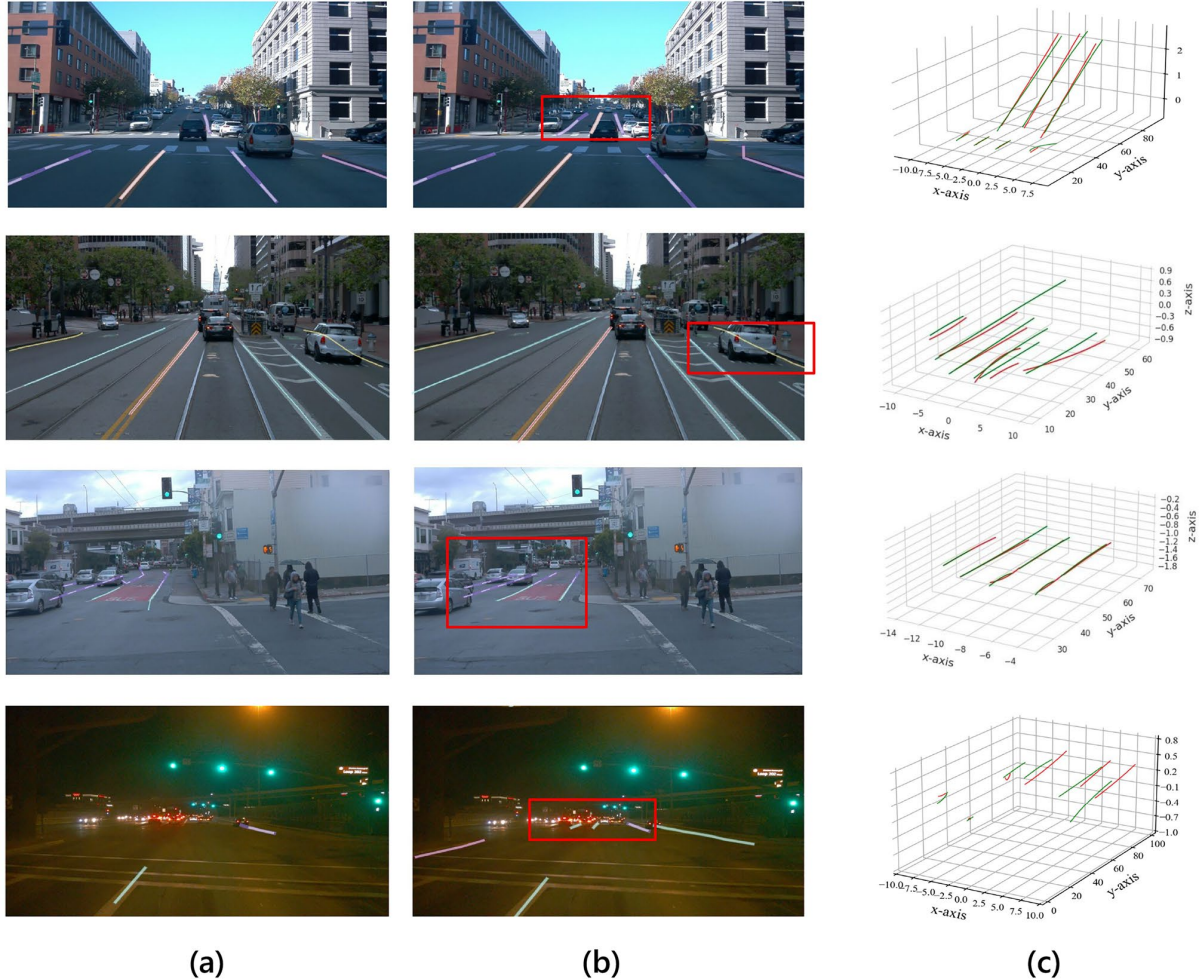
Qualitative Results. The columns illustrate prediction from (**a**) Anchor3DLane ^5^ and (**b**) our method, respectively. Here, different colors indicate specific categories. Column (**c**) demonstrates the ground truth (red) and prediction of CM-3DLane (green) in 3D space.

To further demonstrate the robustness of CM-3DLane, we conducted a dedicated qualitative analysis on challenging scenarios involving severe occlusion, complex shadows, and extreme lighting conditions, as depicted in Fig. 10

In scenarios with heavy vehicle occlusion, baseline methods (Anchor3DLane) often fail to maintain lane continuity, resulting in fragmented detections. In contrast, our LAMamba block, leveraging its global receptive field via the Diagonal Snake Scan, effectively infers the trajectory of occluded lanes by aggregating long-range contextual cues from visible segments.Under strong tree shadows or night-time low-light conditions, traditional CNNs struggle with false positives due to local texture confusion. Our CSAF module plays a critical role here by adaptively enhancing channel-wise features and suppressing noise, allowing the model to distinguish true lane markings from shadow boundaries.

Additionally, we performed a detailed breakdown of F1 scores across different scenarios within the OpenLane dataset, as shown in Table 3. Our method consistently outperformed previous approaches, particularly in challenging environments such as intersections, extreme weather, and night-time conditions. This enhanced performance can be attributed to our method’s improved capability to capture spatial relationships and effectively generalize across diverse environmental conditions. By addressing limitations found in prior methods and refining feature extraction through our model, our approach delivers a more robust and precise solution for monocular 3D lane detection in real world driving scenarios.

**Table 3:** Comparison with other 3D lane detection methods on OpenLane dataset under different scenarios.

Method	All	UP&Down	Curve	Extreme Weather	Night	Intersection	Merge&Split
3DLaneNet ^4^	44.1	40.8	46.5	47.5	43.5	32.1	41.7
Gen-LaneNet ^37^	32.3	25.4	33.5	28.1	18.7	21.4	31.0
PersFormer ^65^	50.5	42.4	55.6	48.6	46.6	40.4	50.7
CurveFormer ^40^	50.5	45.2	56.6	49.7	49.1	42.9	45.4
Anchor3DLane ^5^	53.1	47.2	58.0	51.9	47.2	44.2	50.5
Ours	58.3	50.2	62.9	54.2	53.1	50.3	53.7

A particularly noteworthy aspect of our CM-3DLane framework is its exceptional computational efficiency alongside state-of-the-art detection accuracy. As detailed in Table 4, our model, built upon a lightweight ResNet-18 backbone, achieves the highest inference throughput among all competing methods, running at 78 FPS on a single NVIDIA RTX 2080 Ti GPU with an input size of 360x480. This significantly outperforms the Transformer-based PersFormer (21 FPS) and is also faster than the baseline Anchor3DLane (85 FPS). This speed advantage is attained with the fewest parameters (10.34M) and the lowest computational cost (29.16 GFLOPs) reported in the comparison, which are 13.95% and 23.88% lower than those of Anchor3DLane, respectively. The high FPS, combined with minimal memory footprint and low FLOPs, conclusively demonstrates that CM-3DLane successfully bridges the critical gap between high detection performance and practical deployment efficiency. Its design is particularly well-suited for resource-constrained, real-time autonomous driving systems where both accuracy and latency are paramount.

**Table 4:** Comparison of the complexity of our method with other SOTA methods.

Method	Backbone	F1 (%)	FLOPs	Param (M)	FPS
PersFormer ^65^	EffNet-B7	50.5	572.4G	54.9	21
Anchor3DLane ^5^	ResNet-18	53.1	38.31G	12.2	85
Anchor3DLane ^5^	ResNet-50	53.7	42.4G	13.2	73
Ours	ResNet-18	58.3	29.16G	10.34	78

To comprehensively evaluate the practical deployment efficiency of CM-3DLane, we conducted inference speed benchmarks across various hardware platforms. As shown in Table 5, the tests cover a high-performance desktop GPU (RTX 4090), the original experimental platform used in this paper (RTX 2080 Ti), and an edge computing device targeted for embedded deployment (NVIDIA Jetson AGX Orin). All tests were performed under the conditions of a batch size of 1 and a fixed input resolution of 360$\times$480, using the OpenLane validation set. We report the average per-frame inference time and its FPS.

On the RTX 4090, benefiting from its powerful computing capability, CM-3DLane achieves an extremely high throughput of approximately 159 FPS. On the RTX 2080 Ti, primarily used in this paper, it maintains real-time performance (78 FPS) comparable to lightweight CNN baselines, while delivering superior accuracy in Table 4 . Most importantly, on the resource-constrained Jetson AGX Orin edge platform (running in 30W mode), our method still maintains real-time processing capability above 30 FPS, with a peak GPU memory usage below 1.5 GB. This fully demonstrates that CM-3DLane not only possesses superior algorithmic efficiency (lowest parameters and FLOPs, see Table 4) but its lightweight design also ensures excellent practical deployment performance across hardware with different computational capabilities, meeting the diverse real-time requirements from servers to vehicle-mounted edge devices.

**Table 5:** Inference Efficiency of CM-3DLane on Different Hardware Platforms.

Hardware Platform	Inference Time (ms)	FPS	Peak GPU Memory (GB)
NVIDIA RTX 2080 Ti	10.5	78	1.8
NVIDIA GeForce RTX 4090	6.3	158.7	1.8
NVIDIA Jetson AGX Orin (30W)	33.2	30.1	1.5

### Ablation study

#### Ablation study of components

To quantitatively evaluate the contribution of individual components, we conducted comprehensive ablation experiments on the OpenLane benchmark. As shown in Table 6, we analyze the isolated and combined impacts of CSAF, LAMamba, and RADR on both accuracy and efficiency.

**Effect of Multi-scale Fusion and Global Context**: Individually, the CSAF module improves the F1 score by +2.5% (from 54.8% to 57.3%), demonstrating its effectiveness in aggregating fine-grained texture cues across scales, which is critical for detecting faint lane markings. Similarly, the LAMamba block alone yields a +2.8% gain, validating that modeling long-range spatial dependencies with linear complexity significantly enhances spatial awareness in complex road geometries. When combined, these modules achieve a synergistic effect (+3.1% over baseline), indicating that high-quality multi-scale features are essential prerequisites for the State Space Model to capture global topology effectively.

**Efficiency-Accuracy Trade-off via RADR**: A critical concern in module integration is the computational overhead. While adding CSAF and LAMamba increases FLOPs slightly, it reduces inference speed from 85 FPS to 76 FPS. However, introducing our RADR module reverses this trend. By dynamically pruning redundant anchors based on semantic priors, RADR reduces the computational load in the regression head, recovering the inference speed to 78 FPS while further boosting accuracy to 58.3% F1. Notably, this is achieved with only a marginal parameter increase (from 9.0M to 10.34M). This result conclusively proves that our framework is not a simple stacking of heavy modules, but a *co-designed architecture* where RADR actively optimizes the efficiency bottleneck introduced by enhanced feature extraction.

**Fig. 11:**
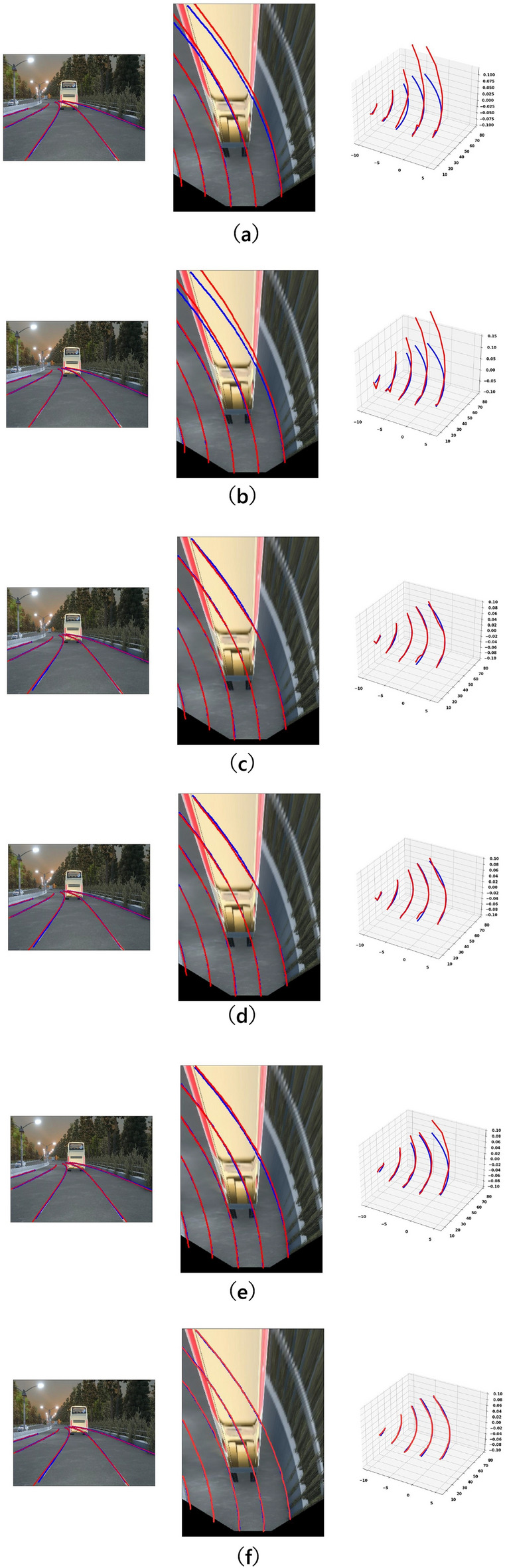
Qualitative ablation study on the effectiveness of key components. As shown: (**a**) Prediction using only the baseline Backbone (**b**) Prediction using only the CSAF module (**d**) Prediction using only the LAMamba block (**e**) Prediction using only the RADR module (**f**) CM-3DLane.

Figure 11 presents a qualitative ablation study on the effectiveness of key components.

**Table 6:** Performance gain for parts of CM-3DLane on OpenLane300 using CSAF, LAMamba, and RADR. We include a baseline and efficiency metrics (FPS) to demonstrate the trade-off between accuracy and speed.

CSAF	LAMamba	RADR	F1 (%)	x-Err/N (m)	x-Err/F (m)	z-Err/N (m)	z-Err/F (m)	FLOPs (G)	Param (M)	FPS
$\times$	$\times$	$\times$	54.8	0.285	0.295	0.225	0.380	24.50	7.50	85
$\checkmark$	$\times$	$\times$	57.3	0.251	0.258	0.201	0.352	27.75	8.00	82
$\times$	$\checkmark$	$\times$	57.6	0.245	0.249	0.200	0.310	26.11	8.00	79
$\checkmark$	$\checkmark$	$\times$	57.9	0.242	0.245	0.198	0.285	28.08	9.00	76
${\checkmark }$	${\checkmark }$	${\checkmark }$	58.3	0.240	0.249	0.196	0.268	26.50	10.34	78

*Note: Baseline is Anchor3DLane. The RADR module recovers inference speed (FPS) by reducing anchor redundancy, offsetting the computational cost of CSAF and LAMamba*

#### Ablation studies on the internal design of the LAMamba block

The scanning path in SS2D determines how spatial contexts are serialized and modeled. To validate our design, we compared our Diagonal Snake Scan against standard strategies (Parallel, Snake, and Diagonal) in Table 7. Standard Parallel Scan achieves the lowest performance (56.1% F1) because its row-by-row processing disrupts the continuity of curved lanes, failing to capture diagonal dependencies. While Snake Scan improves continuity, it still struggles with long-range diagonal curves common in highway exits. Our Diagonal Snake Scan outperforms all baselines, achieving the lowest far-range error (x-Err/F: 0.249m). This confirms our hypothesis: aligning the scanning trajectory with the natural geometric evolution of lane markings (which often extend diagonally in the front view) minimizes the “context breaking” effect, allowing the Mamba block to maintain coherent state transitions over long distances.

**Table 7:** Ablation study results of different SS2D scanning strategies on the OpenLane validation set.

Scanning Strategy	F1 (%)	x-Err/N (m)	x-Err/F (m)	z-Err/N (m)	z-Err/F (m)	FPS
Parallel Scan	56.1	0.255	0.275	0.202	0.350	81
Snake Scan	57.8	0.247	0.253	0.199	0.272	79
Diagonal Scan	56.9	0.250	0.261	0.200	0.315	80
Diagonal Snake Scan (Ours)	58.3	0.240	0.249	0.196	0.268	78

We further investigated the sensitivity of the LAMamba stacking depth (*L*) in Table 8. Increasing *L* from 1 to 3 yields consistent gains in F1 and localization accuracy, suggesting that deeper layers are necessary to encode complex topological structures (e.g., merges and splits). However, increasing *L* to 4 provides a negligible accuracy gain (+0.1%) while causing a significant latency drop (-11.5% FPS, from 78 to 69). This demonstrates the law of diminishing returns in depth scaling for lane tasks. Thus, $L=3$ is empirically determined as the optimal trade-off point, ensuring real-time performance without sacrificing representational capacity.

**Table 8:** Sensitivity analysis of the LAMamba stacking depth (*L*). $L=3$ offers the optimal trade-off between accuracy and latency.

#Layers (*L*)	F1 (%)	x-Err/F (m)	z-Err/F (m)	FLOPs (G)	FPS
1	56.5	0.268	0.121	26.84	92
2	57.6	0.257	0.113	28.00	84
3 (Ours)	58.3	0.249	0.109	29.16	78
4	58.4	0.248	0.110	30.32	69

## Conclusion

In this paper, we propose CM-3DLane, a simple yet effective monocular 3D lane detection framework. We integrate a Cross-Scale Attention Fusion module that dynamically integrates multi-scale features via dual spatial-channel attention pathways; The Lane-Aware Mamba block achieves linear-complexity modeling of long-range lane dependencies while preserving critical texture details; and The Refined Anchor Dynamic Ranking mechanism optimizes anchor selection through geometric priors and attention weighting. Comprehensive evaluations on ApolloSim and OpenLane benchmarks establish new state-of-the-art performance, with our method outperforming existing approaches by significant margins. Notably, CM-3DLane maintains exceptional efficiency (78 FPS) with minimal parameters and computational overhead , demonstrating particular suitability for real-time autonomous systems. Ablation analyses quantitatively verify the complementary benefits of each component, with the complete framework showing marked improvements in handling diverse road scenarios including curves, intersections, and adverse weather conditions. The proposed solution effectively bridges the critical gap between detection accuracy and computational practicality in 3D lane perception. However, the proposed CM-3DLane framework relies on an externally pre-trained 2D lane segmentation model (LaneNet) within its RADR module to provide semantic priors. While this effectively improves anchor selection accuracy, it partially compromises the purely end-to-end nature of the entire system and introduces additional model dependency. Future work will explore deriving similar semantic guidance directly from the multi-scale features of the main network to achieve fully end-to-end joint optimization.

## Data Availability

The ApolloSim and OpenLane datasets analyzed during the current study are publicly available. The ApolloSim dataset can be accessed at https://github.com/yuliangguo/3D_Lane_Synthetic_Dataset. The OpenLane dataset can be accessed at https://github.com/OpenDriveLab/OpenLane.

## References

[1] Altché, F., La Fortelle, A. An lstm network for highway trajectory prediction. In: 2017 IEEE 20th International Conference on Intelligent Transportation Systems (ITSC), pp. 353–359 (2017). IEEE

[2] Zou, Q., Sun, Q., Chen, L., Nie, B. & Li, Q. A comparative analysis of lidar slam-based indoor navigation for autonomous vehicles. *IEEE Trans. Intell. Transp. Syst.* **23**(7), 6907–6921 (2021).

[3] Ma, F., Qi, W., Zhao, G., Zheng, L., Wang, S., Liu, Y., Liu, M. & Ma, J. Monocular 3d lane detection for autonomous driving: Recent achievements, challenges, and outlooks. IEEE Transactions on Intelligent Transportation Systems (2025)

[4] Garnett, N., Cohen, R., Pe’er, T., Lahav, R. & Levi, D. 3d-lanenet: end-to-end 3d multiple lane detection. In: Proceedings of the IEEE/CVF International Conference on Computer Vision, pp. 2921–2930 (2019)

[5] Huang, S., Shen, Z., Huang, Z., Ding, Z.-h., Dai, J., Han, J., Wang, N. & Liu, S. Anchor3dlane: Learning to regress 3d anchors for monocular 3d lane detection. In: Proceedings of the IEEE/CVF Conference on Computer Vision and Pattern Recognition, pp. 17451–17460 (2023)

[6] Targ, S., Almeida, D. & Lyman, K. Resnet in resnet: Generalizing residual architectures. arXiv preprint arXiv:1603.08029 (2016)

[7] Tan, M., Le, Q. Efficientnet: Rethinking model scaling for convolutional neural networks. In: International Conference on Machine Learning, pp. 6105–6114 (2019). PMLR

[8] Yu, F., Koltun, V. Multi-scale context aggregation by dilated convolutions. arXiv preprint arXiv:1511.07122 (2015)

[9] Zhang, H. et al. Robust semantic segmentation for automatic crack detection within pavement images using multi-mixing of global context and local image features. *IEEE Trans. Intell. Transp. Syst.* **25**(9), 11282–11303 (2024).

[10] Dosovitskiy, A., Beyer, L., Kolesnikov, A., Weissenborn, D., Zhai, X., Unterthiner, T., Dehghani, M., Minderer, M., Heigold, G. & Gelly, S., et al. An image is worth 16x16 words: Transformers for image recognition at scale. arXiv preprint arXiv:2010.11929 (2020)

[11] Li, Y. et al. Efficientformer: Vision transformers at mobilenet speed. *Adv. Neural Inform. Process. Syst.* **35**, 12934–12949 (2022).

[12] Zhu, L., Liao, B., Zhang, Q., Wang, X., Liu, W. & Wang, X. Vision mamba: Efficient visual representation learning with bidirectional state space model. arXiv preprint arXiv:2401.09417 (2024)

[13] Liu, Y. et al. Vmamba: Visual state space model. *Adv. Neural. Inf. Process. Syst.* **37**, 103031–103063 (2024).

[14] Gu, A., Goel, K. & Ré, C. Efficiently modeling long sequences with structured state spaces. arXiv preprint arXiv:2111.00396 (2021)

[15] Jin, D., Park, W., Jeong, S.-G., Kwon, H. & Kim, C.-S. Eigenlanes: Data-driven lane descriptors for structurally diverse lanes. In: Proceedings of the IEEE/CVF Conference on Computer Vision and Pattern Recognition, pp. 17163–17171 (2022)

[16] Liu, R., Yuan, Z., Liu, T. & Xiong, Z. End-to-end lane shape prediction with transformers. In: Proceedings of the IEEE/CVF Winter Conference on Applications of Computer Vision, pp. 3694–3702 (2021)

[17] Tabelini, L., Berriel, R., Paixao, T.M., Badue, C., De Souza, A.F. & Oliveira-Santos, T. Polylanenet: Lane estimation via deep polynomial regression. In: 2020 25th International Conference on Pattern Recognition (ICPR), pp. 6150–6156 (2021). IEEE

[18] Pan, X., Shi, J., Luo, P., Wang, X. & Tang, X. Spatial as deep: Spatial cnn for traffic scene understanding. In: Proceedings of the AAAI Conference on Artificial Intelligence, vol. 32 (2018)

[19] Yang, J., Zhang, L. & Lu, H. Lane detection with versatile atrousformer and local semantic guidance. *Pattern Recogn.* **133**, 109053 (2023).

[20] Aly, M. Real time detection of lane markers in urban streets. In: 2008 IEEE Intelligent Vehicles Symposium, pp. 7–12 (2008). IEEE

[21] He, Y., Wang, H. & Zhang, B. Color-based road detection in urban traffic scenes. *IEEE Trans. Intell. Transp. Syst.* **5**(4), 309–318 (2004).

[22] Kim, Z. Robust lane detection and tracking in challenging scenarios. *IEEE Trans. Intell. Transp. Syst.* **9**(1), 16–26 (2008).

[23] Wang, Y., Teoh, E. K. & Shen, D. Lane detection and tracking using b-snake. *Image Vis. Comput.* **22**(4), 269–280 (2004).

[24] Zhou, S., Jiang, Y., Xi, J., Gong, J., Xiong, G. & Chen, H. A novel lane detection based on geometrical model and gabor filter. In: 2010 IEEE Intelligent Vehicles Symposium, pp. 59–64 (2010). IEEE

[25] Hou, Y., Ma, Z., Liu, C. & Loy, C.C. Learning lightweight lane detection cnns by self attention distillation. In: Proceedings of the IEEE/CVF International Conference on Computer Vision, pp. 1013–1021 (2019)

[26] Neven, D., De Brabandere, B., Georgoulis, S., Proesmans, M. & Van Gool, L. Towards end-to-end lane detection: An instance segmentation approach. In: 2018 IEEE Intelligent Vehicles Symposium (IV), pp. 286–291 (2018). IEEE

[27] Qin, Z., Wang, H. & Li, X. Ultra fast structure-aware deep lane detection. In: European Conference on Computer Vision, pp. 276–291 (2020). Springer

[28] Ko, Y. et al. Key points estimation and point instance segmentation approach for lane detection. *IEEE Trans. Intell. Transp. Syst.* **23**(7), 8949–8958 (2021).

[29] Qu, Z., Jin, H., Zhou, Y., Yang, Z. & Zhang, W. Focus on local: Detecting lane marker from bottom up via key point. In: Proceedings of the IEEE/CVF Conference on Computer Vision and Pattern Recognition, pp. 14122–14130 (2021)

[30] Wang, J., Ma, Y., Huang, S., Hui, T., Wang, F., Qian, C. & Zhang, T. A keypoint-based global association network for lane detection. In: Proceedings of the IEEE/CVF Conference on Computer Vision and Pattern Recognition, pp. 1392–1401 (2022)

[31] Xu, S., Cai, X., Zhao, B., Zhang, L., Xu, H., Fu, Y. & Xue, X. Rclane: Relay chain prediction for lane detection. In: European Conference on Computer Vision, pp. 461–477 (2022). Springer

[32] Li, X., Li, J., Hu, X. & Yang, J. Line-cnn: End-to-end traffic line detection with line proposal unit. *IEEE Trans. Intell. Transp. Syst.* **21**(1), 248–258 (2019).

[33] Liu, L., Chen, X., Zhu, S. & Tan, P. Condlanenet: a top-to-down lane detection framework based on conditional convolution. In: Proceedings of the IEEE/CVF International Conference on Computer Vision, pp. 3773–3782 (2021)

[34] Benmansour, N., Labayrade, R., Aubert, D. & Glaser, S. Stereovision-based 3d lane detection system: a model driven approach. In: 2008 11th International IEEE Conference on Intelligent Transportation Systems, pp. 182–188 (2008). IEEE

[35] Bai, M., Mattyus, G., Homayounfar, N., Wang, S., Lakshmikanth, S.K. & Urtasun, R. Deep multi-sensor lane detection. In: 2018 IEEE/RSJ International Conference on Intelligent Robots and Systems (IROS), pp. 3102–3109 (2018). IEEE

[36] Efrat, N., Bluvstein, M., Oron, S., Levi, D., Garnett, N. & Shlomo, B.E. 3d-lanenet+: Anchor free lane detection using a semi-local representation. arXiv preprint arXiv:2011.01535 (2020)

[37] Guo, Y., Chen, G., Zhao, P., Zhang, W., Miao, J., Wang, J. & Choe, T.E. Gen-lanenet: A generalized and scalable approach for 3d lane detection. In: European Conference on Computer Vision, pp. 666–681 (2020). Springer

[38] Liu, R., Chen, D., Liu, T., Xiong, Z. & Yuan, Z. Learning to predict 3d lane shape and camera pose from a single image via geometry constraints. In: Proceedings of the AAAI Conference on Artificial Intelligence, vol. 36, pp. 1765–1772 (2022)

[39] Yan, F., Nie, M., Cai, X., Han, J., Xu, H., Yang, Z., Ye, C., Fu, Y., Mi, M.B. & Zhang, L. Once-3dlanes: Building monocular 3d lane detection. In: Proceedings of the IEEE/CVF Conference on Computer Vision and Pattern Recognition, pp. 17143–17152 (2022)

[40] Bai, Y., Chen, Z., Fu, Z., Peng, L., Liang, P. & Cheng, E. Curveformer: 3d lane detection by curve propagation with curve queries and attention. arXiv preprint arXiv:2209.07989 (2022)

[41] Luo, Y., Zheng, C., Yan, X., Kun, T., Zheng, C., Cui, S. & Li, Z. Latr: 3d lane detection from monocular images with transformer. In: Proceedings of the IEEE/CVF International Conference on Computer Vision, pp. 7941–7952 (2023)

[42] Luo, Y., Cui, S. & Li, Z. Dv-3dlane: End-to-end multi-modal 3d lane detection with dual-view representation. arXiv preprint arXiv:2406.16072 (2024)

[43] Zheng, Z., Zhang, X., Mou, Y., Gao, X., Li, C., Huang, G., Pun, C.-M. & Yuan, X: Pvalane: Prior-guided 3d lane detection with view-agnostic feature alignment. In: Proceedings of the AAAI Conference on Artificial Intelligence, vol. 38, pp. 7597–7604 (2024)

[44] Mehta, S. & Rastegari, M. Mobilevit: Light-weight, general-purpose, and mobile-friendly vision transformer. In: International Conference on Learning Representations (ICLR) (2022)

[45] Liu, H., Fu, C. & Gu, J., et al. Efficientvit: Lightweight multi-scale attention for high-resolution aerospace and mobile vision. In: Proceedings of the IEEE/CVF Conference on Computer Vision and Pattern Recognition (CVPR) (2023)

[46] Fu, D.Y., Dao, T., Saab, K.K., Thomas, A.W., Rudra, A. & Ré, C. Hungry hungry hippos: Towards language modeling with state space models. arXiv preprint arXiv:2212.14052 (2022)

[47] Gu, A. et al. Combining recurrent, convolutional, and continuous-time models with linear state space layers. *Adv. Neural. Inf. Process. Syst.* **34**, 572–585 (2021).

[48] Gu, A., Johnson, I., Timalsina, A., Rudra, A. & Ré, C. How to train your hippo: State space models with generalized orthogonal basis projections. arXiv preprint arXiv:2206.12037 (2022)

[49] Gupta, A., Gu, A. & Berant, J. Diagonal state spaces are as effective as structured state spaces. *Adv. Neural. Inf. Process. Syst.* **35**, 22982–22994 (2022).

[50] Gu, A., Goel, K., Gupta, A. & Ré, C. On the parameterization and initialization of diagonal state space models. *Adv. Neural. Inf. Process. Syst.* **35**, 35971–35983 (2022).

[51] Smith, J.T., Warrington, A. & Linderman, S.W. Simplified state space layers for sequence modeling. arXiv preprint arXiv:2208.04933 (2022)

[52] Gu, A. & Dao, T. Mamba: Linear-time sequence modeling with selective state spaces. arXiv preprint arXiv:2312.00752 (2023)

[53] Wang, C., Tsepa, O., Ma, J. & Wang, B. Graph-mamba: Towards long-range graph sequence modeling with selective state spaces. arXiv preprint arXiv:2402.00789 (2024)

[54] Ma, J., Li, F. & Wang, B. U-mamba: Enhancing long-range dependency for biomedical image segmentation. arXiv preprint arXiv:2401.04722 (2024)

[55] Li, K., Li, X., Wang, Y., He, Y., Wang, Y., Wang, L. & Qiao, Y. Videomamba: State space model for efficient video understanding. In: European Conference on Computer Vision, pp. 237–255 (2024). Springer

[56] Hu, V.T., Baumann, S.A., Gui, M., Grebenkova, O., Ma, P., Fischer, J. & Ommer, B. Zigma: A dit-style zigzag mamba diffusion model. In: European Conference on Computer Vision, pp. 148–166 (2024). Springer

[57] Muksimova, S., Umirzakova, S., Baltayev, J. & Cho, Y.-I. Rl-cervix. net: a hybrid lightweight model integrating reinforcement learning for cervical cell classification. Diagnostics 15(3), 364 (2025) 10.3390/diagnostics15030364 PMC11816595 39941293

[58] Woo, S., Park, J., Lee, J.-Y. & Kweon, I.S. Cbam: Convolutional block attention module. In: Proceedings of the European Conference on Computer Vision (ECCV), pp. 3–19 (2018)

[59] Liu, M., Dan, J., Lu, Z., Yu, Y., Li, Y. & Li, X. Cm-unet: Hybrid cnn-mamba unet for remote sensing image semantic segmentation. arXiv preprint arXiv:2405.10530 (2024)

[60] Liu, H., Yang, J., Miao, X., Mertz, C. & Kong, H. Crackformer network for pavement crack segmentation. *IEEE Trans. Intell. Transp. Syst.* **24**(9), 9240–9252 (2023).

[61] Wang, Z., Ren, W. & Qiu, Q. Lanenet: Real-time lane detection networks for autonomous driving. arXiv preprint arXiv:1807.01726 (2018)

[62] Chen, L., Sima, C., Li, Y., Zheng, Z., Xu, J., Geng, X., Li, H., He, C., Shi, J. & Qiao, Y. et al. Persformer: 3d lane detection via perspective transformer and the openlane benchmark. In: European Conference on Computer Vision, pp. 550–567 (2022). Springer

[63] Sun, P., Kretzschmar, H., Dotiwalla, X., Chouard, A., Patnaik, V., Tsui, P., Guo, J., Zhou, Y., Chai, Y. & Caine, B. et al. Scalability in perception for autonomous driving: Waymo open dataset. In: Proceedings of the IEEE/CVF Conference on Computer Vision and Pattern Recognition, pp. 2446–2454 (2020)

[64] He, K., Zhang, X., Ren, S. & Sun, J. Deep residual learning for image recognition. In: Proceedings of the IEEE Conference on Computer Vision and Pattern Recognition, pp. 770–778 (2016)

[65] Chen, L., Sima, C., Li, Y., Zheng, Z., Xu, J., Geng, X., Li, H., He, C., Shi, J. & Qiao, Y., et al. Persformer: 3d lane detection via perspective transformer and the openlane benchmark. In: European Conference on Computer Vision, pp. 550–567 (2022). Springer

[66] Huang, S., Shen, Z., Huang, Z., Liao, Y., Han, J., Wang, N. & Liu, S. Anchor3dlane++: 3d lane detection via sample-adaptive sparse 3d anchor regression. IEEE Transactions on Pattern Analysis and Machine Intelligence (2024) 10.1109/TPAMI.2024.3508798 40030330

